# Association of particulate matter pollution and case fatality rate of COVID-19 in 49 Chinese cities

**DOI:** 10.1016/j.scitotenv.2020.140396

**Published:** 2020-11-01

**Authors:** Ye Yao, Jinhua Pan, Weidong Wang, Zhixi Liu, Haidong Kan, Yang Qiu, Xia Meng, Weibing Wang

**Affiliations:** a School of Public Health, Fudan University, Shanghai, China; b Sichuan University, Chengdu, China; c Key Laboratory of Public Health Safety of Ministry of Education, Fudan University, Shanghai, China

**Keywords:** COVID-19, Particulate matter pollution, CFR, Cross-sectional study

## Abstract

The COVID-19 epidemic, caused by the SARS-CoV-2 virus, has resulted in 3352 deaths in China as of April 12, 2020. This study aimed to investigate the associations between particulate matter (PM) concentrations and the case fatality rate (CFR) of COVID-19 in 49 Chinese cities, including the epicenter of Wuhan. We used the Global Moran's I to analyze spatial distribution and autocorrelation of CFRs, and then we used multivariate linear regression to analyze the associations between PM_2.5_ and PM_10_ concentrations and COVID-19 CFR. We found positive associations between PM pollution and COVID-19 CFR in cities both inside and outside Hubei Province. For every 10 μg/m^3^ increase in PM_2.5_ and PM_10_ concentrations, the COVID-19 CFR increased by 0.24% (0.01%–0.48%) and 0.26% (0.00%–0.51%), respectively. PM pollution distribution and its association with COVID-19 CFR suggests that exposure to such may affect COVID-19 prognosis.

## Highlights

• Spatial analysis showed a high-high death rate clustering located in Hubei Province.
• Particulate matter (PM) pollution was positively associated with COVID-19 CFR.
• The positive associations happened in cities both inside and outside Hubei Province.
• Results suggested that exposure to PM may affect COVID-19 prognosis.

## Graphical abstract

**Figure:**
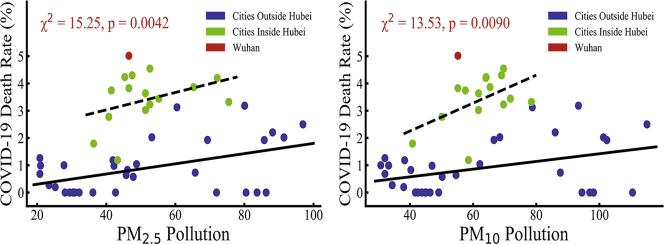

## 1 Introduction

SARS-CoV-2 is a newly emerged coronavirus that has posed immense challenges to global health and has caused unpredictable economic loss. First reported in December of 2019 in Wuhan, China, the Coronavirus Disease 2019 (COVID-19) epidemic exhibits human-to-human transmissibility and has spread rapidly across countries (Li et al., 2020). As of April 12, 2020, a total of 1,610,909 COVID-19 cases and 99,690 deaths have been confirmed in 211 countries, with China reporting a total of 82,214 confirmed cases and 3349 deaths. On March 11th, the World Health Origination (WHO) labelled the COVID-19 outbreak as a global pandemic.

A recent study focused on COVID-19 epidemic in Italy has proposed the hypothesis that air pollution might exacerbate negative prognosis of COVID-19 (Conticini et al., 2020). Exposure to ambient particulate matter (PM) pollution has been reported to increase the risks of mortality and morbidity ofcardiopulmonary diseases worldwide (Atkinson et al., 2014; Chen et al., 2017; Samet et al., 2000; Samoli et al., 2005), with higher likelihood of adverse effects among elderly or people with underlying medical conditions (Zeka et al., 2006); while elderly and people with underlying medical conditions also experienced higher fatality from COVID-19 (Guan et al., 2020; Pan et al., 2020). Toxicological evidence showed that PM could cause pulmonary inflammation and affect the defense system against infection (Donaldson et al., 2001). In addition, exposure to PM could increase inflammation and oxide stress, then aggravate respiratory symptoms and result in increased hospital emergency visits of patients with asthma and chronic obstructive pulmonary disease (COPD) (EPA, 2020). Moreover, air pollution, especially PM pollution, is positively associated with case fatality from other coronavirus infection including severe acute respiratory syndrome (SARS) (Cui et al., 2003). Other studies have proposed that PM could carry viruses as carrier and spread viruses everywhere as a vector (Alonso et al., 2015; Yang et al., 2011).

To date, few studies have estimated and quantified the effects of air pollutants on case fatality rate (CFR) of COVID-19. Therefore, this study aims to investigate the associations between PM_2.5_ and PM_10_ and CFR of COVID-19 in Chinese cities.

## 2 Methods

### 2.1 Data collection

Information on confirmed COVID-19 cases and deaths in China was obtained from the National Health Commission and the Provincial Health Commissions. We calculated the CFR, which was defined as cumulative death counts divided by cumulative confirmed cases, for 49 Chinese cities: Wuhan (capital of Hubei Province), 15 other cities inside Hubei Province, and 33 cities outside Hubei Province. No less than 100 cases of COVID-19 were confirmed in these cities as of March 22, 2020, and few confirmed cases and deaths were reported afterward in China.

Hourly data of PM_2.5_ and PM_10_ concentrations were published by the National Urban Air Quality Publishing Platform (http://106.37.208.233:20035/), a platform administered by China's Ministry of Environmental Protection. Daily average concentrations of PM_2.5_ and PM_10_ were calculated from hourly concentrations for days with at least 18 hourly measurements from each monitoring station. City-wide average concentrations of PM_2.5_ and PM_10_ were calculated based on data from all monitoring sites within the city. Two PM indicators were used in this study, including 1) average PM concentrations from January 15, 2020 to February 29, 2020 to represent the mean PM levels during the main period of COVID-19 in China, and 2) average concentrations of PM annual mean levels in years 2015–2019 to represent the long-term PM levels in these Chinese cities. Gross Domestic Product (GDP) per capita, hospital beds and population size were obtained from statistical yearbook of corresponding provinces (http://data.stats.gov.cn/easyquery.htm). Meteorological data including daily mean temperature and relative humidity at city level were collected from the China Meteorological Data Sharing Service System (http://data.cma.cn/).

### 2.2 Statistical analysis

Spatial auto-correlation statistics have been commonly used to examine spatial dependence or auto-correlation in spatial data. Spatial auto-correlation includes 1) the global spatial auto-correlation which is used for estimating the overall degree of spatial auto-correlation for spatial data, and 2) the local indicators of spatial association (LISA) which is used to assess the influence of individual locations on the magnitude of the global statistic and to identify the locations and types of clusters. The spatial weights were created by rook contiguity rule, and applied to describe the spatial relationships among cities. We explored the spatial distribution of CFRs from 49 cities in China by calculating the Global Moran's I and LISA using ArcToolbox of ArcMap (version 10.2). The calculation formula of Global Moran's I is shown as following:

(1) $$I=\frac{n\cdot \sum_{i=1}^{n}\sum_{j=1}^{n}w_{\mathrm{ij}}\left(x_{i}-\bar{x}\right)\left(x_{j}-\bar{x}\right)}{\left(\sum_{i=1}^{n}\sum_{j=1}^{n}w_{\mathrm{ij}}\right)\sum_{i=1}^{n}{\left(x_{i}-\bar{x}\right)}^{2}},i\neq j$$

LISA is computed as follows:

(2) $$I_{\mathrm{i}}=\frac{\sum_{j=1}^{n}w_{\mathrm{ij}}\left(x_{i}-\bar{x}\right)\left(x_{j}-\bar{x}\right)}{\frac{1}{n}\sum_{i=1}^{n}{\left(x_{i}-\bar{x}\right)}^{2}},i\neq j$$

where *I* is the Global Moran's I, *n* is the number of cities, *x*_*i*_ and *x*_*j*_ are the values of the COVID-19 CFRs of cities *i* and *j*, respectively. $\bar{x}$ is equal to the average of the COVID-19 CFRs of all cities, and *W*_*ij*_ is the spatial weight matrix corresponding to the cities pair *i* and *j*. Global Moran's I index is between −1.0 and 1.0. Moran's I > 0 represents positive spatial correlation, and the greater the value, the more obvious the spatial correlation. Moran's I < 0 represents negative spatial correlation, and the smaller the value, the greater the spatial difference. Otherwise, Moran's I = 0, the space is in random mode. The positively spatial correlation means that the correlation becomes more and more significant with the location (distance) aggregation, and vice versa.

Then, we conducted a cross-sectional analysis to examine the associations of PM_2.5_ and PM_10_ concentrations with the CFRs of COVID-19 in China using multivariate linear regression method, with adjustment for GDP per capita and hospital beds per capita in the main model. As the epicenter of China, the epidemics of COVID-19 occurs earlier in Hubei Province when the disease is not well understood. Therefore, dramatically high number of confirmed cases, high CFR and severe shortage of medical supplies, beds and workers have been encountered in Hubei province. On the other hand, there are huge differences in control policies inside and outside Hubei Province, which could impact the treatment and prognosis of COVID-19 patients, and potentially affect the associations between environmental factors and COVID-19. Additionally, results of spatial cluster analysis also suggested a high-high (HH) CFR clustering located in Hubei Province. Therefore, a two-stage analysis was employed in this study. We first fitted multivariate linear regression models for cities in and out of Hubei Province separately, then pooled the results with meta-analysis. We quantified the percentage changes and 95% confidence intervals (CIs) of CFRs of COVID-19 for every 10 μg/m^3^ increase of PM_2.5_ and PM_10_ concentrations.

In sensitivity analyses, local LISA map values, city size, population number and proportion of people older than 65 years at provincial level was added in multivariate linear regression model for cities outside Hubei Province, respectively, to test the stability of the estimates of PM-CFR associations.

## 3 Results

**Fig. 1:**
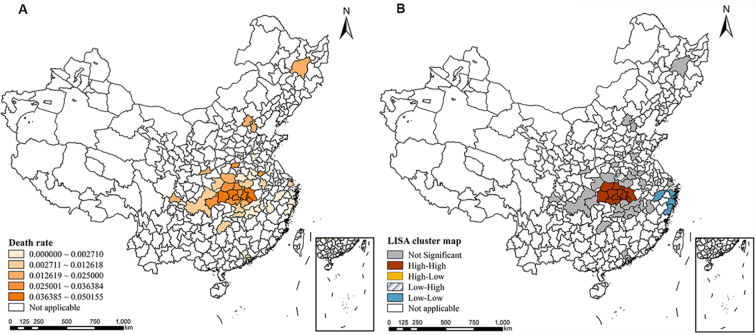
Spatial distribution and LISA cluster of COVID-2019 CFR in 49 Chinese cities with more than 100 confirmed cases.

As of March 22, 2020, a total of 3206 COVID-19 deaths have been reported in the 49 study cities. Of the 3206 deaths, 2508 (78.23%) were reported in Wuhan, Hubei Province, 636 (19.84%) in other cities within Hubei Province, and 62 (1.93%) in other cities outside of Hubei Province (Fig. 1A). As of March 22, the mean CFR of COVID-19 was 1.7% (range: 0.00%–5.00%) among the 49 cities in China.

**Fig. 2:**
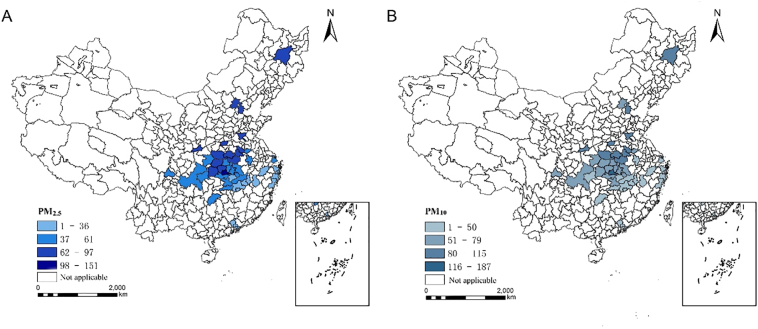
Spatial distribution of PM concentration in 49 Chinese cities with more than 100 confirmed cases.

The value of Global Moran's I was 0.16 (*p* < 0.0001), which indicated a significantly positive global spatial auto-correlation of COVID-2019 CFR in these 49 Chinese cities. Fig. 1B illustrates the distribution of local indicators of spatial association (LISA) map, which is indicative of a high-high (HH) CFR clustering located in Hubei Province. However, Enshi and Shiyan, the two Hubei cities which were farther away from Wuhan, did not fall into the HH cluster. The low-high (LH) CFR cluster included Zhumadian of Henan province. The analysis also showed the core “cold spot” cluster of low-low (LL) districts located in Hangzhou, Ningbo, Taizhou and Wenzhou. Spatial distribution of PM concentration in these 49 Chinese cities was shown in Fig. 2 based on PM levels during the main epidemic period of COVID-19 in China. The mean ± standard deviation was 51.2 ± 20.9 μg/m^3^ (range: 20.8–96.9 μg/m^3^) and 62.1 ± 22.6 μg/m^3^ (range: 30.8–115.1 μg/m^3^) for PM_2.5_ and PM_10_ during the main epidemic period, respectively; and was 49.1 ± 11.0 μg/m^3^ (range: 22.6–73.3 μg/m^3^) and 80.2 ± 20.1 μg/m^3^ (range: 35.6–132.8 μg/m^3^) for average of annual mean concentrations of PM_2.5_ and PM_10_ in 2015–2019, respectively The PM levels in the epidemics period and in long-term (average of annual mean during 2015–2019) were significantly and highly correlated and the correlation coefficients were 0.87 (*p* < 0.001) for PM_2.5_ and 0.85 (*p* < 0.001) for PM_10._

**Fig. 3:**
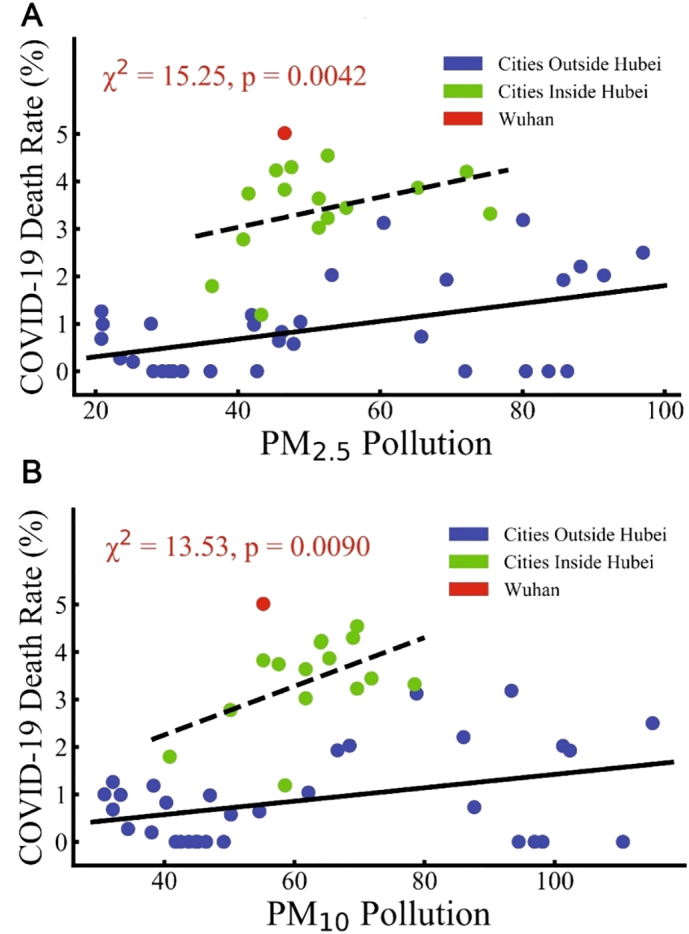
CFR versus PM_2.5_ & PM_10_ pollution. A: CFR was positively associated with PM_2.5_ in cities outside Hubei (blue points, *r* = 0.56, *p* = 0.005) and those inside Hubei except Wuhan (green points, *r* = 0.33, *p* = 0.26) pollution. GDP per capita and hospital beds per capita effects were removed during statistical analysis. B: CFR was positively associated with PM_10_ in cities outside Hubei (blue points, *r* = 0.48, *p* = 0.019) and those inside Hubei except Wuhan (green points, *r* = 0.47, *p* = 0.11) pollution. GDP per capita and hospital beds per capita effects were adjusted during statistical analysis. Average PM concentrations were calculated from January 15, 2020 to February 29, 2020 to represent the mean PM levels during the main period of COVID-19 in China. (For interpretation of the references to colour in this figure legend, the reader is referred to the web version of this article.)

There was no significant difference between cities outside and inside Hubei both in PM_2.5_ (*t* = 0.06, *p* = 0.95) or PM_10_ (*t* = 0.01, *p* = 0.99) in the main epidemic period. COVID-19 CFRs for other Hubei cities was significantly lower than that of Wuhan (*t* = 6.70, *p* < 0.001), while CFRs for cities outside of Hubei was even lower than those inside Hubei (*t* = 8.45, *p* < 0.001). After adjustment for GDP per capita and hospital beds per capita, COVID-19 CFR was positively associated with PM_2.5_ (Fig. 3A, χ^2^ = 15.25, *p* = 0.0042) and PM_10_ (Fig. 3B, χ^2^ = 13.53, *p* = 0.0090) during epidemic period in China. For every 10 μg/m^3^ increase in PM_2.5_ and PM_10_, the CFR increased by 0.24% (0.01%–0.48%) and 0.26% (0.00%–0.51%), respectively (Table 1). The risk estimates increased to 0.61% (0.09% - 1.12%) and 0.33% (0.03% - 0.64%) with every 10 μg/m^3^ increase in average concentrations of PM_2.5_ and PM_10_ in 2015–2019, respectively. In addition, we did not find significance in the association between COVID-19 CFR and GDP per capita (χ^2^ = 4.59, *p* = 0.33), hospital beds per capita (χ^2^ = 8.83, *p* = 0.07), temperature (χ^2^ = 4.22, *p* = 0.38) or relative humidity (χ^2^ = 8.97, *p* = 0.06), respectively. Our previous study suggested that none of temperature, relative humidity or UV irradiance had association with transmissibility and incidence of COVID-19 (Yao et al., 2020). In sensitivity analysis, increased risks of COVID-19 CFR didn't change significantly after adding local LISA map values, city size and population or proportion of people older than 65 years (Table 2).

**Table 1:** Percentage change (mean and 95% CIs)^a^ in COVID-19 CFRs per 10 μg/m^3^ increase in PM_2.5_ and PM_10_ concentrations in Chinese Cities.

Exposure period	Domain	PM_2.5_	PM_10_
Epidemic period	Cities outside Hubei	0.25% (0.10% - 0.40%)	0.20% (0.05% - 0.35%)
	Cities inside Hubei^b^	0.23% (−0.20% - 0.67%)	0.38% (−0.10% - 0.86%)
	Pooled estimates	0.24% (0.01% - 0.48%)	0.26% (0.00% - 0.51%)
Long-term (2015–2019)	Cities outside Hubei	0.61% (0.28% - 0.94%)	0.30% (0.11% - 0.49%)
	Cities inside Hubei^b^	0.60% (−0.32% - 1.52%)	0.41% (−0.15% - 0.98%)
	Pooled estimates	0.61% (0.09% - 1.12%)	0.33% (0.03% - 0.64%)

a The estimates have been adjusted by GDP per capita and hospital beds per capita.

b Cities in Hubei except Wuhan.

**Table 2:** Percentage change (mean and 95% CIs)^a^ in COVID-19 CFRs per 10 μg/m^3^ increase in PM_2.5_ and PM_10_ concentrations in Cities outside Hubei Province with adjustment of local Lisa map values, city size and population, COVID-19 morbidity or proportion of people older than 65 years.

	PM_2.5_^b^	PM_10_^b^
Main model^a^	0.25% (0.10% - 0.40%)	0.20% (0.05% - 0.35%)
Adjust for local Lisa map values^a^	0.18% (0.05% - 0.30%)	0.13% (0.01% - 0.25%)
Adjust for city size and population^a^	0.22% (0.10% - 0.34%)	0.19% (0.07% - 0.30%)
Adjust for proportion of people older than 65 years^a^	0.19% (0.05% - 0.33%)	0.14% (0.01% - 0.26%)

a Models also included GDP per capita and hospital beds per capita.

b Average PM concentrations during the main period of COVID-19 in China.

## 4 Discussions

Our results demonstrate that COVID-19 CFR is significantly associated with PM_2.5_ and PM_10_ in 49 Chinese cities. Our findings are consistent with previous studies of SARS (Cui et al., 2003). COVID-19 is caused by SARS-CoV-2, which shares 79.6% sequence identity with SARS-CoV and has the same cell entry receptor-angiotensin converting enzyme II (ACE- II) (Zhou et al., 2020). Limited studies have reported on the associations between air pollution and the SARS epidemic, which lasted for half year in 2003 and only a few Chinese cities reported adequate confirmed and death cases to support further analysis. One previous study indicated that moderate air pollution index (APIs), which was dominated by PM_10_ in China in 2003, was associated with a 84% increased risk of dying from SARS compared to those from regions with low APIs, and SARS patients from regions with high APIs were twice as likely to die from SARS compared to those from regions with low APIs (Cui et al., 2003). Other previous studies demonstrated that exposure to PM_10_ and PM_2.5_ might damage lung functions (Bloemsma et al., 2016; Doiron et al., 2019; Rice et al., 2013). The potential mechanism for PM exposure on respiratory outcomes might be the activation of inflammatory pathways in the small respiratory airways in response to PMs, leading to the recruitment of inflammatory cells (Kelly and Fussell, 2011). These biological mechanisms might potentially influence the prognosis of COVID-19 patients.

In this study, PM-fatality associations are significantly positive no matter if using average PM levels during epidemics period or in 2015–2019. Considering that the patients died from COVID-19 are likely to stay in hospital for treatment without being directly exposed to ambient air pollution prior to death. Therefore, we speculate that the effects of PM_2.5_ and PM_10_ on death mainly affect the progress of patients from mild to severe and prognosis, when the patients were not isolated from ambient air pollution. Additionally, our results showed positive association between long-term PM exposure and COVID-19 CFR, suggesting that long term PM exposure that prior to the epidemics period could have increased vulnerability of population to SARS-CoV-2. The resutls suggested that there is still a need to increase our efforts in the control of air pollutant emissions, which is critical for potential resurgence of COVID-19 epidemics in the future.

This ecological association study is limited by the duration of study period. However, the correlation between COVID-19 CFR and air pollution based on large sample size is noteworthy. Other city-level factors, such as implementation ability of COVID-19 control policy, urbanization rate, and availability of medical resources, might affected the CFR of COVID-19; however, we controlled the GDP per capita and hospital beds per capita to ensure the credibility of the results. Also, the risk estimates stayed stable after adding other covariates including local LISA map values, city size and population or proportion of people older than 65 years. Previous studies reported that some chronic diseases, such as hypertension, diabetes and cardiopulmonary diseases were also potentially linked to the pathogenesis of COVID-19 (Yang et al., 2020); thus the co-morbidities of these diseases may serve as confounders to associations of PM exposure and COVID-19 CFR. The effects of prevalence of these diseases should be investigated further when data available. Limited by the resolution of COVID-19 data, which was only available at city level, exposure assessments of PM_2.5_ and PM_10_ in this study were done at city level based on ground monitoring data rather than using gridded predicting data of PM_2.5_ and PM_10_ at high spatial resolution (*e.g.* 1 km × 1 km), which might cause exposure misclassification. Nevertheless, we believe that this exposure misclassification would not substantially bias our findings since: 1) the ground monitoring sites were normally located at areas of high population density in China that could reflect the exposure levels of the majority of residents; 2) and this kind of nondifferential misclassification may lead to underestimates on the effects of PM_2.5_ and PM_10_ (Goldman et al., 2011). In addition, based on publicly available data, it is not yet possible to obtain the number of COVID-19 cases and deaths of different ages in all cities to further investigate the modification of age and other medical conditions on PM-fatality associations.

## CRediT authorship contribution statement

**Ye Yao:** Conceptualization, Methodology, Formal analysis, Investigation, Writing - original draft. **Jinhua Pan:** Formal analysis, Investigation, Writing - original draft. **Weidong Wang:** Formal analysis, Investigation. **Zhixi Liu:** Formal analysis, Investigation, Writing - original draft. **Haidong Kan:** Formal analysis, Investigation, Writing - review & editing. **Yang Qiu:** Writing - original draft, Writing - review & editing. **Xia Meng:** Conceptualization, Methodology, Writing - review & editing, Formal analysis. **Weibing Wang:** Conceptualization, Methodology, Writing - review & editing.

## Declaration of competing interest

The authors declare that they have no known competing financial interests or personal relationships that could have appeared to influence the work reported in this paper.

## Appendix A Supplementary data

Supplementary Table 1 City size and population, number of COVID-19 confirmed and dead cases, and fatality rate of COVID-19 for 49 Chinese Cities. Supplementary Table 1
